# Honokiol Inhibits Non-Small Cell Lung Cancer Cell Migration by Targeting PGE_2_-Mediated Activation of β-Catenin Signaling

**DOI:** 10.1371/journal.pone.0060749

**Published:** 2013-04-08

**Authors:** Tripti Singh, Santosh K. Katiyar

**Affiliations:** 1 Birmingham Veterans Affairs Medical Center, Birmingham, Alabama, United States of America; 2 Department of Dermatology, University of Alabama at Birmingham, Birmingham, Alabama, United States of America; 3 Nutrition Obesity Research Center, University of Alabama at Birmingham, Birmingham, Alabama, United States of America; 4 Comprehensive Cancer Center, University of Alabama at Birmingham, Birmingham, Alabama, United States of America; University of Kentucky, United States of America

## Abstract

Lung cancer remains a leading cause of death due to its metastasis to distant organs. We have examined the effect of honokiol, a bioactive constituent from the *Magnolia* plant, on human non-small cell lung cancer (NSCLC) cell migration and the molecular mechanisms underlying this effect. Using an *in vitro* cell migration assay, we found that treatment of A549, H1299, H460 and H226 NSCLC cells with honokiol resulted in inhibition of migration of these cells in a dose-dependent manner, which was associated with a reduction in the levels of cyclooxygenase-2 (COX-2) and prostaglandin E_2_ (PGE_2_). Celecoxib, a COX-2 inhibitor, also inhibited cell migration. Honokiol inhibited PGE_2_-enhanced migration of NSCLC cells, inhibited the activation of NF-κB/p65, an upstream regulator of COX-2, in A549 and H1299 cells, and treatment of cells with caffeic acid phenethyl ester, an inhibitor of NF-κB, also inhibited migration of NSCLC cells. PGE_2_ has been shown to activate β-catenin signaling, which contributes to cancer cell migration. Therefore, we checked the effect of honokiol on β-catenin signaling. It was observed that treatment of NSCLC cells with honokiol degraded cytosolic β-catenin, reduced nuclear accumulation of β-catenin and down-regulated matrix metalloproteinase (MMP)-2 and MMP-9, which are the down-stream targets of β-catenin and play a crucial role in cancer cell metastasis. Honokiol enhanced: (i) the levels of casein kinase-1α, glycogen synthase kinase-3β, and (ii) phosphorylation of β-catenin on critical residues Ser^45^, Ser^33/37^ and Thr^41^. These events play important roles in degradation or inactivation of β-catenin. Treatment of celecoxib also reduced nuclear accumulation of β-catenin in NSCLC cells. FH535, an inhibitor of Wnt/β-catenin pathway, inhibited PGE_2_-enhanced cell migration of A549 and H1299 cells. These results indicate that honokiol inhibits non-small cell lung cancer cells migration by targeting PGE_2_-mediated activation of β-catenin signaling.

## Introduction

Lung cancer is responsible for more deaths in the US each year than breast, colon and prostate cancers combined, and thus has a tremendous impact on human health and health care expenditures [1]. One of every three cancer-related deaths is attributable to lung cancer, and has no improvement over the last about 30 years [2], [3]. Non-small-cell lung cancer (NSCLC) accounts for approximately 80% of all types of lung cancer and includes adenocarcinoma, squamous cell carcinoma and large-cell carcinomas [4], [5]. Cyclooxygenase-2 (COX-2) is frequently constitutively up-regulated in different human malignancies, including lung cancers [6]–[10]. Although multiple genetic changes are necessary for lung cancer risk and its development, COX-2 is considered as a central element in orchestrating the lung carcinogenesis. COX-2 is an inducible enzyme and generates prostaglandins (PGs) upon its action on arachidonic acid. Among the PGs, PGE_2_ is considered the most effective metabolite or inflammatory mediator that is thought to play a central role in cancer growth, progression, invasion and metastasis. Studies in colon cancer, where COX-2 is spontaneously overexpressed, have revealed a link between COX-2/PGE_2_ and β-catenin signaling which contributes to the growth of colon cancer [11]. Smith et al [12] have shown that ultraviolet radiation-induced COX-2 expression and PGE_2_ production results in enhanced activation of β-catenin signaling. There are reports which suggest that COX-2/PGE_2_/β-catenin axis or link is associated with the lung cancer metastasis [13]. β-catenin is a 90 kD cytosolic protein and acts as a crucial component of the Wnt pathway. In the absence of Wnt ligands, β-catenin is recruited to the phosphorylation/destruction complex, which contains the tumor suppressor, adenomatous polyposis coli (APC) and Axin. The destruction complex facilitates the phosphorylation of β-catenin by glycogen synthase kinase 3β and casein kinase (CK1) leading to the proteasomal degradation of β-catenin. If β-catenin is not phosphorylated then N-terminally un-phosphorylated β-catenin accumulates in cytosol, it enters the nucleus and interacts with transcription factors, such as T-cell factor, to activate transcription of target genes which are associated with cell survival, proliferation and metastasis [14]–[16]. Since, lung cancer is a highly malignant cancer with a potent capacity to metastasize distantly and a major cause of cancer-related deaths, an approach that reduces its metastatic ability may facilitate the development of an effective strategy for its treatment and/or prevention.

**Figure 1:**
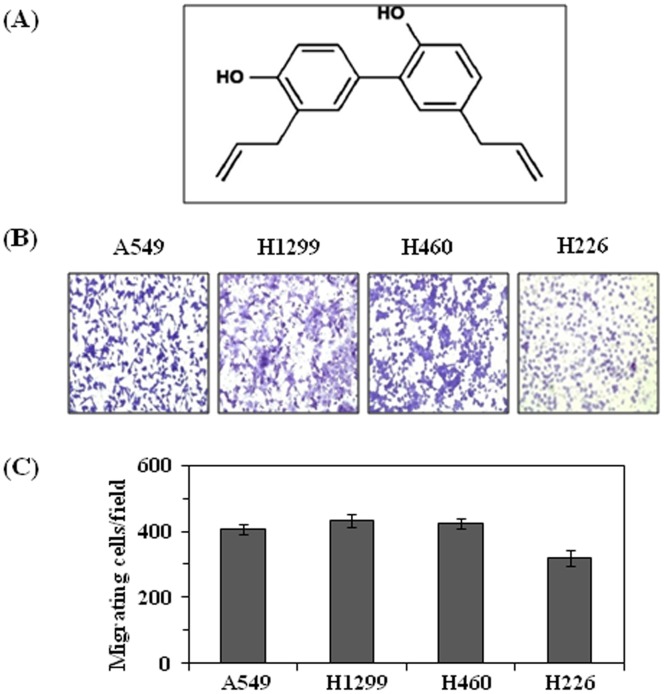
Migration potential of various NSCLC cell lines. (**A**) Molecular structure of honokiol, a bioactive phytochemical from the *Magnolia* plant. (**B**) The cell migration potential of various NSCLC cell lines was determined using a Boyden chamber assay. Equal numbers of cancer cells were loaded in the upper chamber of Boyden chambers, incubated for 24 h, and migratory cells were detected on the membrane after staining with crystal violet. (**C**) The migratory cells were counted and the results are expressed as the mean number of migratory cells ± SD/selected microscopic field, n = 3, magnification: ×10.

Phytochemicals of therapeutic values offer promising options for the development of effective strategies for the prevention of tumor cell migration, invasion and metastasis. Honokiol (C_18_H_18_O_2_, Figure 1A) is a promising bioactive constituent of the bark of *Magnolia* plants that has been used in traditional Japanese medicine for the treatment of some ailments due to its antithrombotic, antidepressant and anti-bacterial properties [17]. Anti-carcinogenic effects of honokiol have been investigated in a variety of cancer cell lines as well as in some tumor models and exhibit no apparent toxicity *in vivo* [18]–[25]. Our studies also have shown that honokiol exerts chemopreventive effects on ultraviolet radiation-induced skin cancer and that this effect is associated with its targeting inflammatory mediators, such as COX-2 and PGE_2_ [25]. However, little is known as to whether honokiol targets invasion or metastatic potential of lung cancer cells. As lung cancer is highly metastatic, we sought to determine the chemotherapeutic effect of honokiol on lung cancer cell migration or invasion using various lung cancer cell lines as an *in vitro* model. In the present communication, we explored the chemotherapeutic effects of honokiol on the migration/invasive potential of human NSCLC cells and ascertained whether inhibitory effect of honokiol on cell migration is associated with the inactivation of the β-catenin signaling and whether PGE_2_ has any role in this process. For this purpose, four different NSCLC cell lines were selected: A549, H1299, H460 and H226. Normal human bronchial epithelial cell line (BEAS-2B) was used as a control. Here, we present evidence that honokiol inhibits the invasive potential of NSCLC cell lines by targeting PGE_2_-mediated activation of β-catenin signaling.

## Materials and Methods

### Reagents and Antibodies

Purified honokiol was purchased from Quality Phytochemicals, LLC (Edison, NJ). Boyden Chambers and polycarbonate membranes (8 µm pore size) for cell migration assays were obtained from Neuroprobe (Gaithersburg, MD). The antibodies specific for β-catenin were purchased from R&D Biosystems (Minneapolis, MN), celecoxib, PGE_2_ were from Sigma Chemical Company, an enzyme immunoassay kit for PGE_2_ analysis was obtained from Cayman Chemicals (Ann Arbor, MI), while antibodies for phospho β-catenin, CK1α, GSK-3β, matrix metalloproteinase (MMP)-2, MMP-9, COX-2, NF-κB, IKKα, IκBα and β-actin were obtained from Cell Signaling Technology (Beverly, MA). Secondary antibodies (rabbit anti-goat and goat anti-rabbit) conjugated with horseradish peroxidase were purchased from Santa Cruz Biotech (Santa Cruz, CA).

### Cell Lines and Cell Culture

NSCLC cell lines, A549, H460, H226 and H1299, and BEAS-2B cell line were obtained from the American Type Culture Collection (Manassas, VA). These cell lines were maintained and cultured as detailed previously [26]. Honokiol was dissolved in a small volume of ethyl alcohol, which was added to the complete cell culture medium when cells were sub-confluent (60–70% confluent). Maximum concentration of ethyl alcohol was not more than 0.02% (v/v) in the culture medium. Cells treated with ethyl alcohol (0.02%, v/v) only served as a vehicle control, as used earlier [27].

### Cell Invasion Assay

The invasion potential of NSCLC cells was determined *in vitro* using Boyden Chambers (Gaithersburg, MD), as described previously [26], [27]. The migrating cells on the surface of the membranes were examined microscopically and cellular invasion was determined by counting the migrating/invasive cells in at least 4–5 randomly selected fields using an Olympus BX41 microscope and photomicrographs were obtained using a Qcolor5 digital camera system fitted to this microscope. Each experiment was repeated two to three times and the resultant cell invasion data are presented in terms of the mean number of invasive or migrating cells ± SD/microscopic field (magnification, ×10).

### Wound Healing or Scratch Assay

Wound healing assay was performed to examine the migration ability of NSCLC cells, as detailed previously [28]. Briefly, NSCLC cells were grown to full confluency in six-well plates and incubated overnight in starvation medium. Cell monolayer was scratched with a sterile fine pipette tip and washed with medium to remove detached cells from the plates. Cells were kept in incubator with or without treatment with honokiol in full culture medium for 36 h. After 36 h, medium was replaced with PBS buffer. The wound gap was observed under Olympus BX41 microscope and cells were photographed using a Qcolor5 digital camera.

### Cell Viability Assay

The effect of honokiol on the proliferative capacity or cell viability of the normal human bronchial epithelial cells and NSCLC cells was determined using 3-(4,5-dimethylthiazol-2-yl)-2,5-diphenyltetrazolium bromide (MTT) assay as described previously [26].

### Prostaglandin E_2_ Immunoassay

Cayman PGE_2_ Enzyme Immunoassay Kit (Ann Arbor, MI) was used to measure the levels of PGE_2_ in cell homogenates following the manufacturer’s instructions, as also described previously [27].

### NF-κB/p65 Activity Assay

The NF-κB/p65 activity was determined using the NF-κB Trans^AM^ Activity Assay Kit (Active Motif, Carlsbad, CA) following the manufacturer’s protocol, and also described previously [27]. The results on NF-κB/p65 activity in cells are expressed as the percentage of the optical density of the non-honokiol-treated control group.

### COX-2 Small Interfering RNA (siRNA) Transfection of NSCLC Cells

A549 and H1299 cell lines were transfected with human-specific COX-2 siRNA using the siRNA Transfection Reagent Kit (Santa Cruz Biotechnology, Inc.; Santa Cruz, CA) following the manufacturer’s protocol. Briefly, 2×10^5^ cells/well were seeded in a 6-well plate and allowed to grow up to 70% confluency. The COX-2 siRNA mix with transfection reagents was overlaid on the cells for approximately 6 h in an incubation chamber and transferred into 2× growth medium for about 20 h. At 24 h post-transfection, fresh medium was added and the cells were incubated for an additional 48 h as detailed previously [29]. Thereafter, cells were harvested and subjected to the cell migration assay. The knockdown of COX-2 expression in A549 and H1299 cells after transfection was verified as described previously [29].

### Western Blot Analysis

NSCLC cells were treated with or without honokiol or other agents of interest for desired time period, thereafter the cells were harvested, and lysed with ice-cold lysis buffer supplemented with protease inhibitors, as described previously [26], [30]. Proteins were electrophoretically resolved on 8–10% Tris-Glycine gels and transferred onto a nitrocellulose membrane. After blocking the non-specific binding sites, the membrane was incubated with the primary antibody at 4°C overnight. Membranes were washed and then incubated with the peroxidase-conjugated secondary antibody and the specific protein bands were detected using the enhanced chemiluminescence reagents. To verify equal loading of proteins on the gels, the membrane was stripped and reprobed with either anti-β actin or anti-histone H3 antibody.

### Statistical Analysis

For cell migration assays, the data in control group were compared with honokiol-, PGE_2_- or celecoxib-treatment groups separately using one-way analysis of variance (ANOVA) using GraphPad Prism version 4.00 for Windows software (GraphPad Software, San Diego, California. www.graphpad.com.). All quantitative data are shown as mean ± SD. In each case *P*<0.05 was considered statistically significant.

## Results

### Comparative Analysis of the Invasive Potential of Human NSCLC Cells

First, we determined the comparative invasive potential of different NSCLC cell lines, such as A549, H1299, H460 and H226, using Boyden chamber assay. Incubation of the NSCLC cells for 24 h in Boyden chamber resulted in a greater number of migration of cells which reflects their invasiveness. Representative photomicrographs of crystal violet-stained cells are shown in Figure 1B. Migratory or invasive cells were counted under microscope, and resultant data are presented in terms of the mean number of invasive cells ± SD/microscopic field (magnification, ×10) (Figure 1C). As shown in Figure 1C, the invasion capacity of cells was almost identical except that the invasive potential of H226 cells was comparatively lower than other cell lines. Under identical conditions, the migration potential of normal human bronchial epithelial cells was significantly lower than NSCLS cells (data not shown).

### Honokiol Inhibits Cell Migration of Human NSCLC Cells

**Figure 2:**
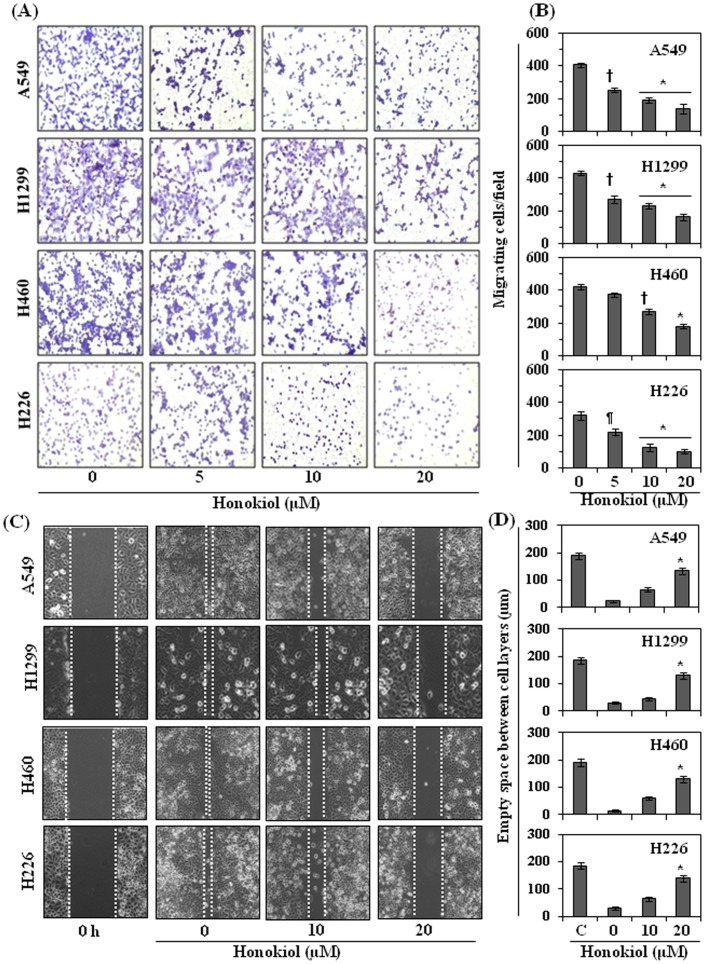
Honokiol inhibits migration potential of NSCLC cells. (**A**) Treatment of NSCLC cells with honokiol (0, 5, 10 and 20 µM) for 24 h inhibits migration of cells in a dose-dependent manner. (**B**) The migrating cells were counted in each treatment group and the results are presented as the mean number of migratory cells ± SD/field, n = 3. Significant inhibition in cell migration *versus* non-honokiol-treated control group, *P<0.001, ^†^ *P<*0.01; ^¶^ *P*<0.05. (**C**) Wound healing or scratch assay was performed to assess the effect of honokiol on the migration ability of NSCLC cells. Cells were incubated with or without honokiol for 36 h. Honokiol inhibits migration of cells in a dose-dependent manner compared to control (non-honokiol-treated) cells. Control (0 h) panel indicates the original space between the cell layers immediate after making a scratch or wound. The space between the broken white lines indicates the space largely unoccupied by the lung cancer cells. The representative photomicrographs are shown from three independent experiments. (**D**) The unoccupied empty space by the cells between the cell layers was measured using micro-grid scale under microscope, and the data are presented as an empty space in terms of µm ±SD for each cell line. Significant inhibition in cell migration versus non-honokiol-treated controls, *P<0.001.

The effect of honokiol was determined on the cell migration or invasive potential of A549, H1299, H460 and H226 human NSCLC cell lines using Boyden chamber cell migration assays. Initially, a screening experiment was performed to determine the effects of lower concentrations of honokiol (µM). As shown in Figure 2A, relative to non-honokiol-treated control cells, treatment of cells with honokiol at concentrations of 0, 5, 10 and 20 µM for 24 h reduced the invasive potential of these cancer cells in a concentration-dependent manner. The density of the migrating cells on the membrane after staining with crystal violet is shown in Figure 2A, and the numbers of migrating cells/microscopic field are summarized in Figure 2B. The cell migration was inhibited by 38 to 66% (*P*<0.01–0.001) in A549 cells, by 37–62% (*P*<0.01–0.001) in H1299 cells, 12–58% (*P*<0.05–0.001) in H460 cells and 32–69% (*P*<0.05–0.001) in H226 cells in a concentration-dependent manner after treatment with honokiol (Figure 2B). Comparatively higher inhibitory effect of honokiol on cell migration was observed at the 48 h time point (data not shown).

**Figure 3:**
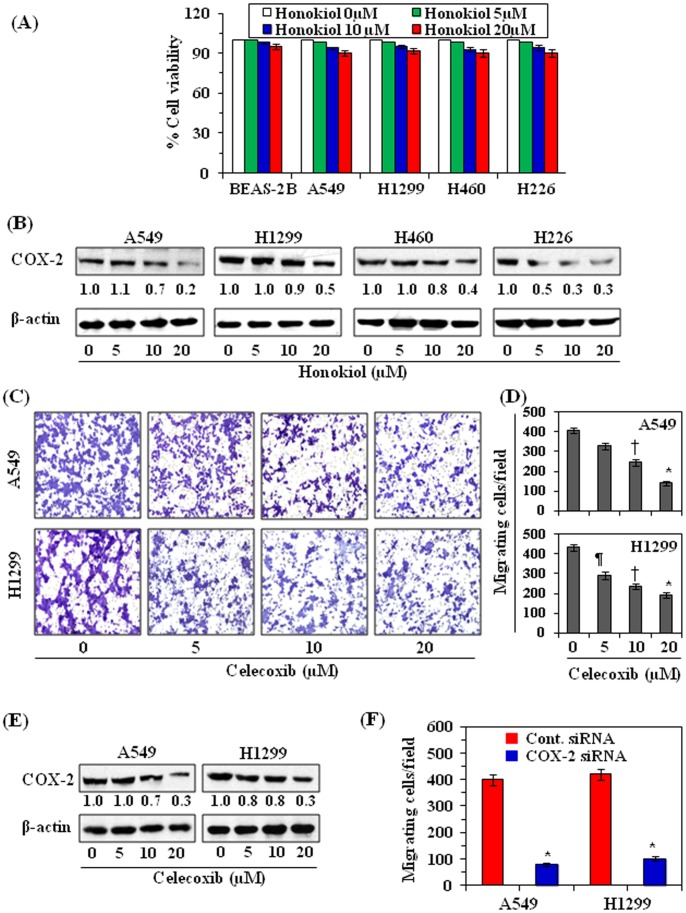
Effect of honokiol on the endogenous expression of COX-2 in NSCLC cells. (**A**) A comparative dose-dependent effect of honokiol on the proliferation potential of BEAS-2B cells and NSCLC cell lines, as analyzed by MTT assay. (**B**) Cells were treated with various concentrations of honokiol for 24 h, and cell lysates were subjected to western blot analysis to measure the levels of COX-2. (**C**) Treatment of A549 and H1299 cells with celecoxib, an inhibitor of COX-2, for 24 h inhibits the cell migration potential in a dose-dependent manner. (**D**) The number of migrating cells was counted on the membrane and the results are expressed as the mean number of migratory cells ± SD/field from three separate experiments. Significant inhibition by honokiol versus non-honokiol-treated control cells, *P<0.001, ^†^ *P*<0.01, ^¶^ *P*<0.05. (**E**) Effect of celecoxib on the expression levels of COX-2 in A549 and H1299 cells. Cells were treated with various concentrations of celecoxib for 24 h, then harvested and cell lysates were subjected to western blot analysis for the measurement of COX-2 levels. The relative density of protein bands in blots was measured using an ImageJ program developed at the National Institutes of Health (http://rsb.info.nih.gov/ij) and normalized with the β-actin bands. In each case control group was assigned an arbitrary unit 1.0. (**F**) Transfection of A549 and H1299 cells with COX-2 siRNA significantly decreases cell migration. Significant reduction of cell migration versus control siRNA-treated cells, *P<0.001.

The inhibitory effect of honokiol on NSCLC cell migration was further verified using a wound healing assay, as described in the Material and Methods. For wound healing assay, the effect of honokiol on cell migration was determined at 36 h after its treatment whereas in Boyden chamber assay the effect of honokiol was determined after 24 h. It was due to the fact that in wound healing assay first cells attached to the plates, then start proliferating and afterwards start migrating or start healing the wounds. Thus, it takes longer time. As shown in Figure 2C, relative to non-honokiol-treated control cells, treatment of cells with honokiol (0, 10 and 20 µM) for 36 h reduced the migration capacity of all cell lines (A549, H1299, H460 and H226) used in this study in a dose-dependent manner. The major gap or wounding space between cell layers after making a wound was covered by the migrating NSCLC cells which were not treated with honokiol. However, the empty space between cell layers was less covered by the migrating cells treated with honokiol and this effect was dose-dependent. The wounding or empty space between the cell layers is highlighted by broken lines (Figure 2C). These data further suggests that honokiol inhibited the migratory efficiency of NSCLC cells. The space between the broken white lines was measured microscopically in each treatment group. As summarized in Figure 2D, the empty space between cell layers was significantly greater in honokiol-treated cells (*P*<0.001) as compared to non-honokiol-treated control cells. This shows that honokiol inhibited the process of cancer cell migration. To verify that the inhibition of cancer cell migration by honokiol was a direct effect on migration ability, and that was not due to a reduction in cell viability, an MTT assay was performed using cells that were treated identically to those used in the migration assays. Treatment of normal human bronchial epithelial cells (BEAS-2B) and NSCLC cells with honokiol at the concentrations of 0, 5, 10 and 20 µM had no significant inhibitory effect on cell viability, as shown in Figure 3A.

### The Inhibitory Effect of Honokiol on the Cell Migration of NSCLC Cells is Associated with the Reduction of Endogenous COX-2 Expression

To examine whether honokiol inhibits NSCLC cell migration by targeting the endogenous expression of COX-2, we determined the levels of COX-2 in lysates of cells treated with and without honokiol using western blot analysis. As shown in Figure 3B, treatment of A549, H1299, H460 and H226 cells with honokiol reduced the levels of COX-2 expression in a concentration-dependent manner as compared to the expression of COX-2 in untreated controls. These results suggest that inhibition of cancer cell migration by honokiol may be associated with the inhibition of COX-2 expression in cancer cells.

### Celecoxib, a Selective COX-2 Inhibitor, Inhibits NSCLC Cell Migration

To determine whether the inhibitory effect of honokiol on NSCLC cell migration is mediated through its inhibitory effect on COX-2 expression, equal numbers of A549 and H1299 cells were subjected to the cell migration assay after treatment with various concentrations of celecoxib (0, 5, 10, 20 µM) for 24 h. As shown in Figure 3C, treatment of the cells with celecoxib resulted in a dose-dependent reduction in the cell migration capacity of these cells as compared with non-celecoxib-treated controls. Data on cell migration are summarized in Figure 3D, which suggested that treatment of cells with celecoxib significantly inhibited (*P*<0.01–0.001) the migration of A549 and H1299 cells in a dose-dependent manner. These data suggested that the inhibition of endogenous constitutive levels of COX-2 expression is associated with the inhibition of NSCLC cell migration. Further, the levels of COX-2 were checked in the lysates of cells treated or non-treated with celecoxib using western blot analysis. Western blot data revealed that treatment of A549 and H1299 cells with celecoxib for 24 h resulted in reduction of COX-2 expression in these cells, as shown in Figure 3E.

### Selective COX-2 Knockdown Using siRNA Leads to Reduction of Cancer Cell Migration

The role of COX-2 in cell migration was further verified using siRNA knock-down of COX-2 in the NSCLC cells and examined whether it would lead to the inhibition of the cell migration in cancer cells. The transfection of A549 and H1299 cells with COX-2 siRNA resulted in significant reduction of cell migration in A549 (79%, *P*<0.001) and H1299 (75%, *P*<0.001) cells after 24 h as compared to the migration of control siRNA-transfected A549 and H1299 cells (Figure 3F).

### Honokiol Inhibits the Production of PGE_2_ and PGE_2_-enhanced Cell Migration in NSCLC Cells

**Figure 4:**
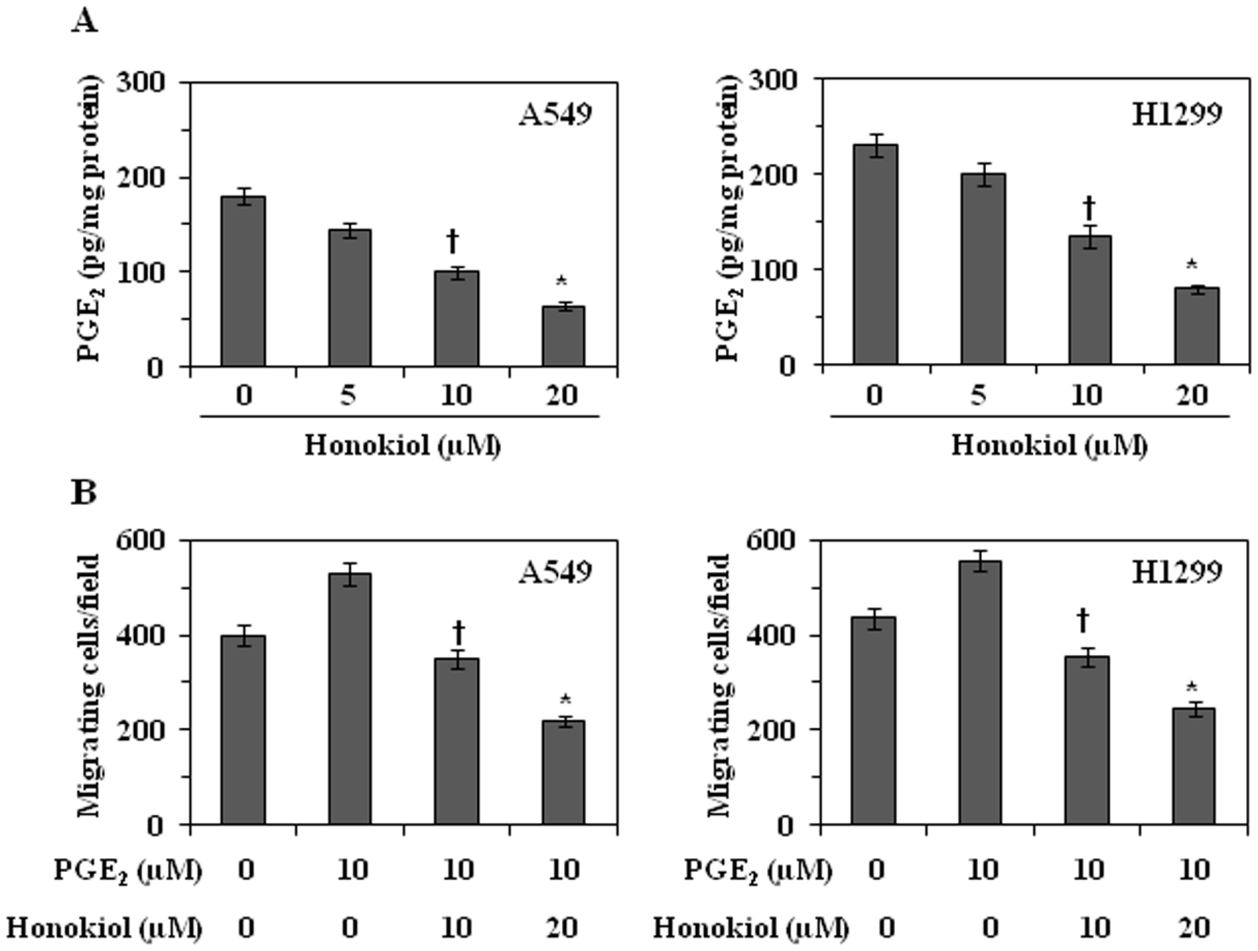
Effect of honokiol on PGE_2_ production and PGE_2_-enhanced migration of NSCLC cells. (**A**) Treatment of A549 and H1299 cells with honokiol reduced the levels of PGE_2_ in a dose-dependent manner. The levels of PGE_2_ were measured in cell lysates using PGE_2_ immunoassay kit and results are expressed in terms of pg/mg protein ± SD, n = 3. Significant reduction versus control cells, *P<0.001, ^†^ *P*<0.01. (**B**) Treatment of A549 and H1299 cells with honokiol inhibits PGE_2_-enhanced cell migration ability. The data on cell migration capacity of cells are summarized as a mean number of migratory cells ± SD/microscopic field, n = 2. Significant inhibition versus PGE_2_ alone treatment; *P<0.001, ^†^ *P*<0.01.

As the chemopreventive effect of honokiol on cell migration ability of 4 different NSCLC cell lines was almost identical, we have selected 2 cell lines (A549 and H1299) for further detailed and mechanistic studies. As PGE_2_ is a major metabolite of COX-2 and has been implicated in COX-2-mediated adverse effects including the invasion and metastasis of cancer cells; we determined the levels of PGE_2_ in the honokiol-treated cells using PGE_2_ immunoassay kit. Our results revealed that treatment of cells with honokiol for 24 h resulted in significant reduction in the production of PGE_2_ in both A549 (20–64%, *P*<0.01–0.001) and H1299 (13–65%, *P*<0.01–0.001) cells in a dose-dependent manner (Figure 4A), suggesting that honokiol-induced reduction in PGE_2_ production may be associated with an inhibitory effect of the honokiol on the cell migration in these cells.

Next, we examined whether PGE_2_ enhanced the migration of NSCLC cells and whether honokiol inhibits PGE_2_-induced cell migration in human NSCLC cells. For this purpose, A549 and H1299 cells were treated with PGE_2_ (10 µM) with and without honokiol for 24 h and cell migration determined. The *in vitro* treatment dose of PGE_2_ was selected on the basis of previous studies [29], [31]. It was found that the treatment of NSCLC cells with PGE_2_ resulted in a significant increase in cell migration (*P*<0.05) compared to the cells which were not treated with PGE_2_ (Figure 4B). Treatment of A549 and H1299 cells with honokiol (10 and 20 µM) resulted in significant inhibition (*P*<0.01–0.001) of PGE_2_ (10 µM)-induced cell migration (Figure 4B).

### Honokiol Inhibits NF-κB/p65 Activity in NSCLC Cells: NF-κB is an Important Regulator of Cancer Cell Invasion/migration

**Figure 5:**
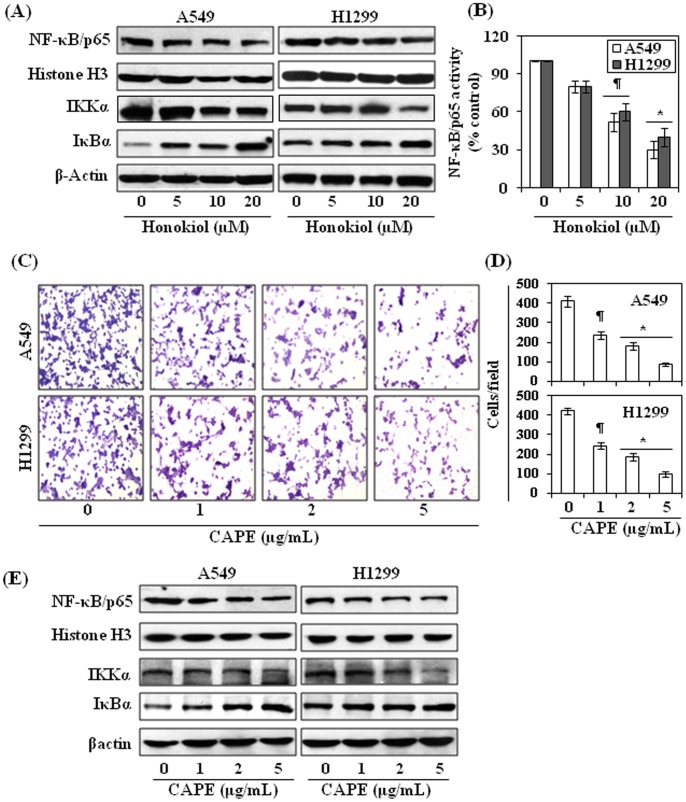
Honokiol inhibits the activation of NF-κB/p65 in NSCLC cells. (**A**) A549 and H1299 cells were treated with varying concentrations of honokiol for 24 h, cells were harvested and cytosolic and nuclear fractions were subjected to western blot analysis. (**B**) The activity of NF-κB/p65 in the nuclear fractions of cells were measured using NF-κB/p65-specific activity assay kit, n = 3. NF-κB/p65 activity is expressed in terms of percent of non-honokiol-treated control cells. Significant inhibition *versus* control, *P<0.001, ^¶^ *P*<0.05. (**C**) Treatment of cells with CAPE, an inhibitor of NF-κB, for 24 h inhibits the migration of cells in a dose-dependent manner. (**D**) Data on cell migration are summarized as the mean number of migrating cells ± SD per microscopic field/group. Significant inhibition *versus* control group, *P<0.001, ^¶^ *P*<0.05. (**E**) Treatment of cells with CAPE for 24 h inhibits the levels of NF-κB and IKKα as determined using western blot analysis.

NF-κB is an up-stream regulator of COX-2; therefore we determined whether honokiol affects the activity as well as the levels of proteins of NF-κB family in NSCLC cells. For this purpose, again A549 and H1299 cells were treated with honokiol (0, 5, 10 and 20 µM) for 24 h, and thereafter cells were harvested and nuclear lysates and whole cell lysates were prepared for western blot analysis. Western blot analysis revealed that treatment of cells with honokiol reduces the nuclear accumulation of NF-κB/p65 in a dose-dependent manner (Figure 5A). The activity of NF-κB/p65 also was significantly reduced (*P*<0.05 and *P*<0.001) after the treatment of cells with honokiol (Figure 5B). Our data also revealed that treatment of honokiol resulted in the suppression of IKKα, an enzyme responsible for NF-κB activation, and prevented degradation of IκBα (Figure 5A). To further verify whether NF-κB has a role in NSCLC cell migration, A549 and H1299 cells were treated with a potent inhibitor of NF-κB, caffeic acid phenethyl ester (CAPE), and cell migration was determined. As shown in Figure 5C and 5D, treatment of cells with CAPE for 24 h resulted in a dose-dependent reduction of cell migration of A549 (42–78%, *P*<0.05–0.001) and H1299 (41–76%, *P*<0.05–0.001) cells relative to non-honokiol-treated control cells, and this effect of CAPE was similar to that observed on treatment of the cells with honokiol (Figures 2A, 2B). Further, we also checked the effect of CAPE on the proteins of NF-κB family in A549 and H1299 cells using western blot analysis. As shown in Figure 5E, treatment of cells with CAPE resulted in the suppression of the levels of NF-κB/p65 and IKKα in both cell lines in a dose-dependent manner.

### Honokiol Inhibits Nuclear Accumulation of β-catenin

**Figure 6:**
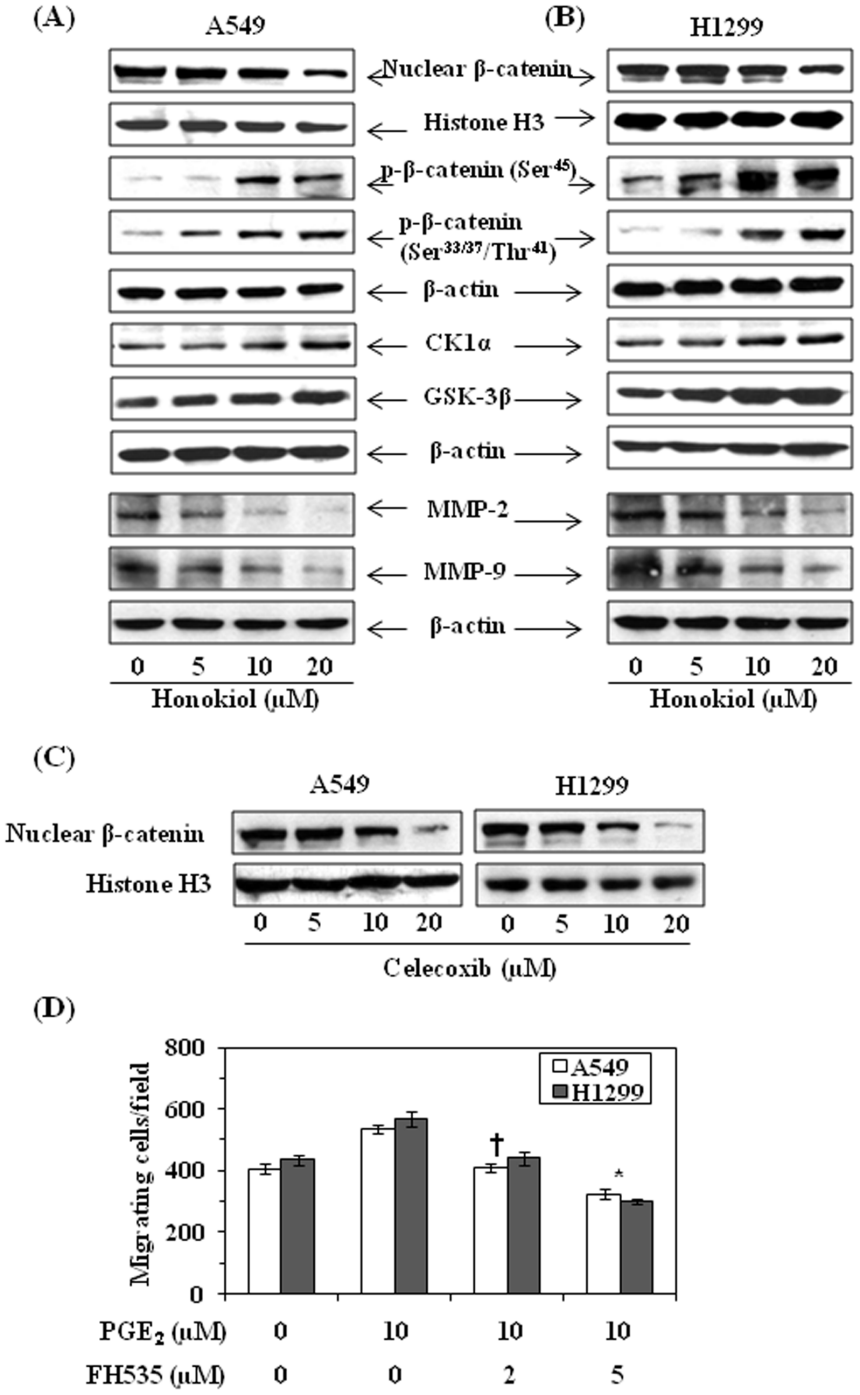
Effect of honokiol on β-catenin and its signaling molecules in NSCLC cells. (**A**) Effect of honokiol on the levels of β-catenin, phosphorylation of β-catenin at critical serine residues, regulatory kinases, such as CK1α and GSK-3β, and MMP-2 and MMP-9 in A549 cells. (**B**) Effect of honokiol on the levels of β-catenin, phosphorylation of β-catenin at critical serine residues, regulatory kinases, such as CK1α and GSK-3β, and MMP-2 and MMP-9 in H1299 cells. (**C**) Treatment of A549 and H1299 cells with celecoxib reduces nuclear accumulation of β-catenin. (**D**) FH535, an inhibitor of Wnt/β-catenin signaling, inhibits PGE_2_-enahnced migration of A549 and H1299 cells. Significant inhibition versus PGE_2_-alone-treated control, *P<0.001, ^†^ *P*<0.01.

PGE_2_ has been shown to activate β-catenin signaling pathway which has been implicated in cancer cell growth, invasion and metastasis [12], [13]. As we have found that treatment of NSCLC cells with honokiol inhibits the levels of PGE_2_ and inhibits PGE_2_-enhanced migration of lung cancer cells, we have further determined whether inhibition of NSCLC cell migration by honokiol is also associated with the suppression of β-catenin signaling. For this purpose, A549 and H1299 cells were treated with honokiol for 24 h, and nuclear and cytosolic fractions of cell lysates were subjected to the analysis of proteins of β-catenin signaling. Western blot analysis revealed that treatment of A549 and H1299 cells with honokiol resulted in reduction of the levels of nuclear β-catenin (Figures 6A and 6B) in a dose-dependent manner. Since, nuclear accumulation of β-catenin is inversely correlated with phosphorylation at certain key residues of β-catenin (Ser^45^, Ser^33^, Ser^37^ and Thr^41^), we checked the effect of honokiol on the levels of β-catenin phosphorylation at these sites. It was observed that treatment of A549 and H1299 cells with honokiol increased the phosphorylation of β-catenin at Ser^45^, and Ser^33^/Ser^37^/Thr^41^ in both NSCLC cell lines (Figures 6A, 6B). Additionally, honokiol treatment of these cells resulted in a dose-dependent increase of CK1α and GSK-3β. Both CK1α and GSK-3β are known to target β-catenin for proteasomal degradation *via* combined phosphorylation at key serine and threonine residues of β-catenin [15]. As MMP-2 and MMP-9 are the downstream targets of β-catenin [32]–[34], and play a role in cancer cell metastasis, we also examined the effect of honokiol on the levels of MMP-2 and MMP-9 in NSCLC cell lines. Consistent with the decreased nuclear accumulation of β-catenin after treating the cells with honokiol, the expression of MMP-2 and MMP-9 were also found to be decreased in both A549 and H1299 cells after treatment of the cells with honokiol (Figures 6A and 6B).

Just to verify whether reduced nuclear accumulation of β-catenin by honokiol is due to its inhibitory effect on COX-2 expression, we treated the cells with celecoxib, an inhibitor of COX-2, for 24 h. Cells were harvested and nuclear lysates were prepared and subjected to the analysis of β-catenin. Western blot analysis revealed that treatment of A549 and H1299 cells with celecoxib resulted in reduced nuclear accumulation of β-catenin compared to non-celecoxib-treated controls (Figure 6C). These data provide support to the evidence that COX-2 and/or PGE_2_ have a role in activation of β-catenin signaling mechanism.

### FH535, an Inhibitor of β-catenin, Inhibits PGE_2_-enhanced Cell Migration of NSCLC Cells

To verify whether activation of β-catenin and PGE_2_ has a role in migration/invasion of NSCLC cells, A549 and H1299 cells were treated with PGE_2_ (10 µM) with and without the treatment with FH535 (2 and 5 µM) for 24 h and cell migration was determined using Boyden Chamber assay. Mean number of migrating cells per microscopic field ± SD in different treatment groups are summarized in Figure 6D. As shown in Figure 6D, treatment of cells with PGE_2_ enhanced the migration ability of cancer cells, and treatment of cells with FH535 significantly inhibited PGE_2_-enhanced migration of lung cancer cells (*P*<0.01–0.001).

## Discussion

It is well known that COX-2 overexpression is found in a wide variety of human cancers, including lung cancer, and is linked to all stages of tumorigenesis as well as cancer cell invasion. COX-2 overexpression is associated with human lung cancer cell-derived PGE_2_ which promotes tumor cell survival, invasion and metastasis [35]. The use of selective COX-2 inhibitors has demonstrated potential in treatment of lung cancer but long-term safety and toxicity concerns have hindered their acceptance as viable clinical chemopreventive agents. Exploration of new agents with low toxicity that target PGE_2_-mediated mechanism of cancer cell migration and/or invasion should lay the basis for chemopreventive strategies that will reduce the risk of lung cancer metastasis and ultimately prevent lung cancer-related human deaths.

The critical findings of the present study are that the treatment of NSCLC cells with honokiol for 24 h inhibits cell migration in a concentration-dependent manner, and that is associated with the inhibition of endogenous expression of COX-2 and production of PGE_2_. Based on our observation, cell death or apoptosis is not a reason of honokiol-induced inhibition of NSCLC cell migration because the concentrations of honokiol used in these experiments are low and are not capable to induce significant inhibitory effect on cell viability at 24 h after treatment. Lung cells overexpress COX-2, and the inhibition of COX-2 by honokiol may contribute to the inhibition of cell migration of lung cancer cells. This concept is supported by the evidence that treatment of the NSCLC cells with celecoxib, a potent COX-2 inhibitor, resulted in a reduction in cell migration. Additionally, honokiol inhibits PGE_2_-enhanced cell migration of A549 and H1299 cells. These observations support the evidence that inhibition of NSCLC cell migration by honokiol requires the inhibition of COX-2 expression and reduction in the production of PGE_2_ metabolite. These findings suggest the feasibility of using honokiol as an alternative to COX-2 inhibitors, which show toxicity in some patients, given the fact that COX-2 remains a promising target for cancer treatment. Similar to honokiol, other phytochemicals also have been investigated and shown to have anti-cancer cell migration activity. Treatment of non-small cell lung cancer cells with proanthocyanidins resulted in inhibition of cell migration following the inhibition of nitric oxide and guanylate cyclase pathways [36]. Epigallocatechin-3-gallate, a polyphenolic constituent of green tea, has been shown to inhibit mammary cancer cell migration through the inhibition of nitric oxide and nitric oxide-mediated mechanisms [37]. Grape seed proanthocyanidins inhibit melanoma cell migration/invasiveness by reduction of PGE_2_ synthesis and reversal of epithelial-to-mesenchymal transition [38].

NF-κB is an upstream regulator of COX-2; therefore, we checked the effect of honokiol on the NF-κB in A549 and H1299 NSCLC cells, and found that treatment of these cells with honokiol decreased the level as well as activity of NF-κB/p65 in a dose-dependent manner and simultaneously down-regulated the levels of IKKα, which is responsible for NF-κB activation. Treatment of NSCLS cells with caffeic acid phenethyl ester, an inhibitor of NF-κB, resulted in an inhibitory effect on lung cancer cell migration. These observations suggest that the inhibitory effect of honokiol on lung cancer cell migration is mediated, at least in part, through the downregulation of COX-2 expression and PGE_2_ production, which are the downstream targets of NF-κB. However, it is possible that down-regulation of other NF-κB target genes could also contribute to the inhibition of NSCLC cell migration.

Similar to honokiol, traditional non-steroidal anti-inflammatory drugs, such as sulindac, inhibit COX-2 expression and resulting in reduced Wnt-signaling by induced β-catenin degradation, as has been shown in colon cancer [39]. Similar to non-steroidal anti-inflammatory drugs, honokiol also induced β-catenin degradation or reduced nuclear accumulation of β-catenin in NSCLC cells and that is associated with inhibition of lung cancer cell migration. Various studies have implicated the role of constitutively active Wnt/β-catenin signaling in tumor progression. β-catenin is a dual function protein and is an important component of cell-to-cell adhesion, where it forms a dynamic link between E-cadherin and cytoskeleton [40], [41]. This cell-to-cell adhesion may prevent the migration of tumor cells. In contrast, the breaking of cell-to-cell adhesion due to activation of β-catenin and its nuclear accumulation may increase the migration potential of tumor cells. It can also regulate cell migration via its role as a transcription factor wherein it regulates expression of various target genes that mediate cellular processes including cell migration [14]. Thus nuclear/cytoplasmic ratio of β-catenin in the cells determines their migration potential. The results from our study show that honokiol inhibits lung cancer cell migration, and this inhibition of cell migration is associated with degradation of cytosolic β-catenin and decreased nuclear accumulation of β-catenin. It has been shown that phosphorylation of β-catenin at critical target residues such as at Ser^45^, Ser^33/37^ and Thr^41^ by GSK-3β and CK1α within the cytosolic destruction complex leads to degradation of β-catenin and that leads to its lesser nuclear accumulation [15]. We have found that treatment of NSCLC cells with honokiol enhances the expressions of CK1α and GSK-3β as well as enhanced phosphorylation of β-catenin at critical serine and threonine target residues. This event of change may lead to degradation of β-catenin within the degradation complex resulting in its reduced nuclear accumulation. This whole event of cascade explains the inhibitory effects of honokiol against non-small cell lung cancer cell migration.

**Figure 7:**
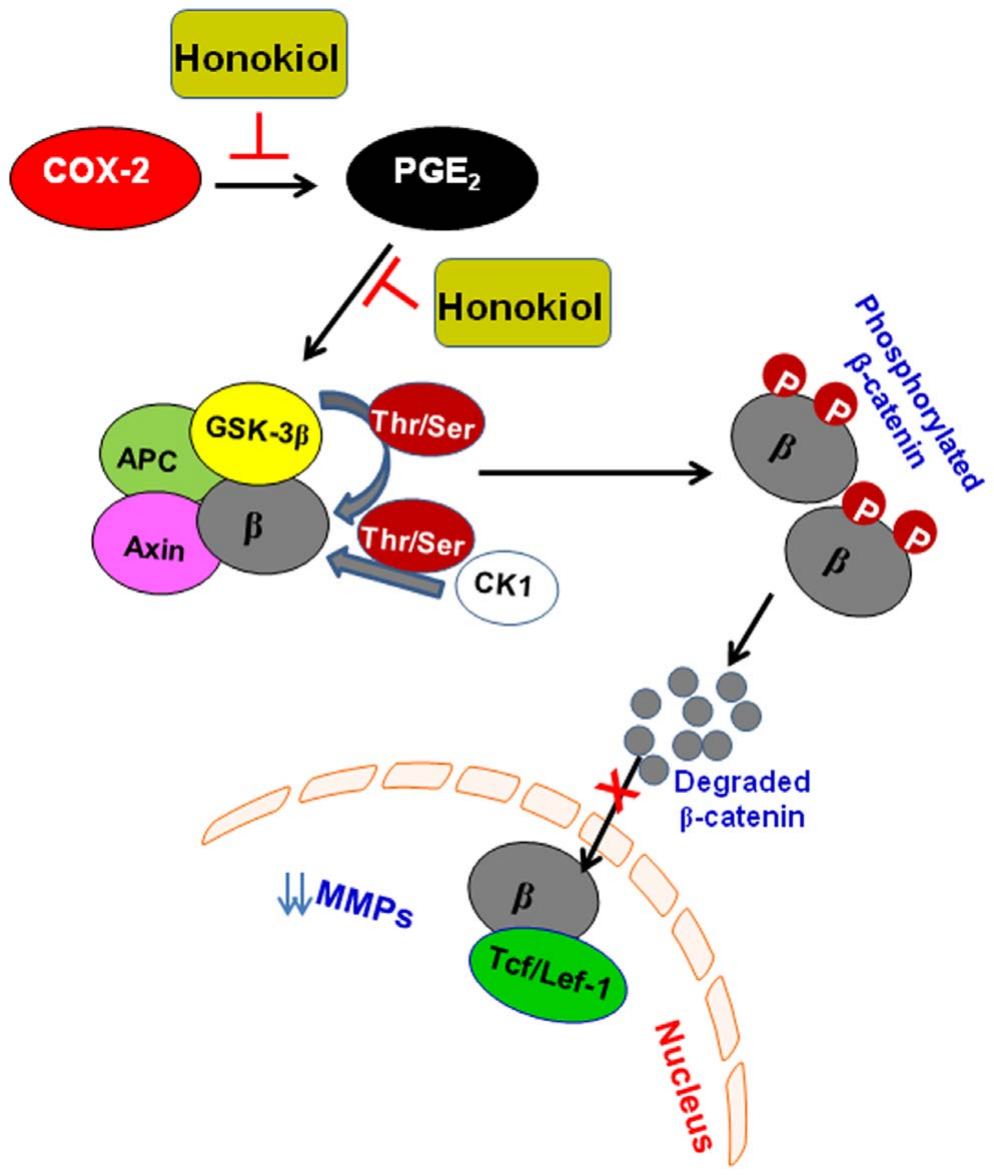
Schematic diagram summarizes the mechanism of action of honokiol on NSCLC cell migration. Honokiol inhibits the endogenous expression of COX-2 and production of PGE_2_ which leads to degradation or inactivation of β-catenin. Degradation of β-catenin in cytosol results in reduction of nuclear accumulation and that leads to inhibition of migration of NSCLC cells.

In an attempt to further establish a link between PGE_2_ and β-catenin in tumor cell migration, and verify the role of honokiol in prevention of NSCLC cell migration through downregulation of PGE_2_ and inactivation of β-catenin signaling, A549 and H1299 cells were treated with PGE_2_ with and without the treatment with FH535, an inhibitor of β-catenin. We found that FH535 reduced PGE_2_-enhanced migration of A549 and H1299 cells. Simultaneously, treatment of cells with celecoxib, an inhibitor of COX-2, reduced the migration of lung cancer cells as well as reduced the nuclear accumulation of β-catenin. Together, these observation support the hypothesis that honokiol inhibits the migration of NSCLC cells by targeting PGE_2_-mediated activation of β-catenin signaling. Together, the results from this study have identified for the first time that honokiol inhibits the migration of NSCLC cells and that this effect involves: (i) the inhibitory effect of honokiol on COX-2 and PGE_2_, (ii) the inhibitory effect of honokiol on NF-κB activation, and (iii) inactivation of β-catenin signaling. These events are summarized in Figure 7. Thus intervention strategies targeting key signaling molecules of the PGE_2_-Wnt/β-catenin pathway may represent promising options to inhibit metastasis of lung cancer cells, and may serve as the basis for chemoprevention or therapy of lung cancer in human patients.
